# Functional Analysis of *O*-GlcNAcylation in Cancer Metastasis

**DOI:** 10.3389/fonc.2020.585288

**Published:** 2020-10-27

**Authors:** Donglu Wu, Jingji Jin, Zhidong Qiu, Da Liu, Haoming Luo

**Affiliations:** ^1^ School of Clinical Medical, Changchun University of Chinese Medicine, Changchun, China; ^2^ Key Laboratory of Effective Components of Traditional Chinese Medicine, Changchun University of Chinese Medicine, Changchun, China; ^3^ School of Pharmacy, Changchun University of Chinese Medicine, Changchun, China

**Keywords:** *O*-GlcNAcylation, *O*-GlcNAc transferase (OGT), cancer metastasis, transcriptional factors, post-translational modifications (PTMs)

## Abstract

One common and reversible type of post-translational modification (PTM) is the addition of *O*-linked β-N-acetylglucosamine (*O*-GlcNAc) modification (*O*-GlcNAcylation), and its dynamic balance is controlled by *O*-GlcNAc transferase (OGT) and glycoside hydrolase *O*-GlcNAcase (OGA) through the addition or removal of *O*-GlcNAc groups. A large amount of research data confirms that proteins regulated by *O*-GlcNAcylation play a pivotal role in cells. In particularly, imbalanced levels of OGT and *O*-GlcNAcylation have been found in various types of cancers. Recently, increasing evidence shows that imbalanced *O*-GlcNAcylation directly or indirectly impacts the process of cancer metastasis. This review summarizes the current understanding of the influence of *O*-GlcNAc-proteins on the regulation of cancer metastasis. It will provide a theoretical basis to further elucidate of the molecular mechanisms underlying cancer emergence and progression.

## Introduction

As one of the post-translational modifications (PTMs), *O*-GlcNAcylation often occurs on serine (Ser) and threonine (Thr) residues of specific substrate cellular proteins including transcription factors, signaling pathway members and metabolic enzymes (1). *O*-GlcNAc transferase (OGT) and *O*-GlcNAcase (OGA) are responsible for adding or removing *O*-GlcNAc groups at the serine/threonine (Ser/Thr) residues of the target proteins to maintain the dynamic balance of intracellular *O*-GlcNAcylation (2). The sugar nucleotide uridine diphospho-N-acetylglucosamine (UDP-GlcNAc) which is generated by the nutrient-dependent hexosamine biosynthetic pathway (HBP), serves as a donor for *O*-GlcNAc addition to specific substrate proteins, demonstrating the link between glucose metabolism and *O*-GlcNAcylation (3). Therefore, *O*-GlcNAcylation is often referred to as a nutrient sensor. It has been found that *O*-GlcNAcylation is involved in diverse fundamental cellular processes, including cell signaling as well as tumorigenesis and tumor progression (4). A decade of research regarding the role of *O*-GlcNAcylation in cancer progression has resulted in accumulating studies on its potential roles in metastasis. Here, the role of *O*-GlcNAcylation in cancer metastasis will be summarized. In addition, the potential roles of the *O*-GlcNAcylation-PTMs axis in metastasis and small molecules that target *O*-GlcNAcylation are discussed.

## OGT and OGA Jointly Maintain Intracellular *O*-GlcNAcylation

### Molecular Structure of OGT and OGA

As mentioned previously, intracellular *O*-GlcNAcylation is dynamically regulated by OGT and OGA. Notably, OGT and OGA are the only enzymes found to be involved in the addition and removal of *O*-GlcNAc groups to or from Ser/Thr residues of the substrate proteins (5). Human cells express three isoforms of OGT—nucleocytoplasmic (ncOGT, 116kDa), mitochondrial (mOGT, 103kDa), and short (sOGT, 75kDa)—which differ only in their subcellular location and number of N-terminal tetratricopeptide-repeats (TPRs), three different transcripts contain 13.5 (ncOGT), 9 (mOGT), and 3 (sOGT) TPRs, respectively. It is already clear that OGT is divided into two highly conserved functional domains (**Table 1**) (6, 7, 14). The N-terminal TPR domain binds the substrate protein, while the C-terminal catalytic domain binds UDP-GlcNAc and catalyzes *O*-GlcNAcylation of the substrate (8, 15–17). And OGA was initially isolated from crude cellular extract, and it catalyzes hydrolytic cleavage of *O*-GlcNAc from proteins (18). There are two alternative OGA splicing isoforms as follows: OGA-L (916 amino acids) predominantly localizes in the cytoplasm, and OGA-S (677 amino acids) localizes to the nucleus and lipid-droplets (8, 15–17, 19). OGA is also divided into two functional domains, N-terminus N-acetyl-β-D-glucosaminidase domain and C-terminal pseudo-histone acetyltransferase (HAT) domain (20). In cells, OGA can interact with OGT to form an “O-GlcNAczyme” complex under high glucose conditions (21), however disrupting this balance will lead to abnormal cell function and possibly even cancer.

**Table 1:** Isoforms and functional characteristics of OGT.

Isoforms	Location	TPRs	Functions	Self-*O*-GlcNAc modification sites	Reference
ncOGT	****Nucleus and cytoplasm****	13.5****	*O*-GlcNAcylates the nucleus, cytoplasm and mitochondrial proteins	Ser10, Thr12, Ser20, Thr38, Ser52, Ser56, Ser389, Ser437, Thr662	(6–10),
mOGT	Mitochondria****	9****	Maintains the structure and function of mitochondria	–	(8, 9, 11, 12),
sOGT	Nucleus and**** cytoplasm****	3****	Self-*O*-GlcNAc modification	Ser10, Thr12, Ser18, Thr38	(7–9, 13),

*ncOGT, nucleocytoplasmic O-GlcNAc transferase; mOGT, mitochondrial O-GlcNAc transferase; sOGT, short O-GlcNAc transferase; Ser, Serine; Thr, Threonine.

### Imbalanced *O*-GlcNAcylation in Cancer Cells

**Figure 1:**
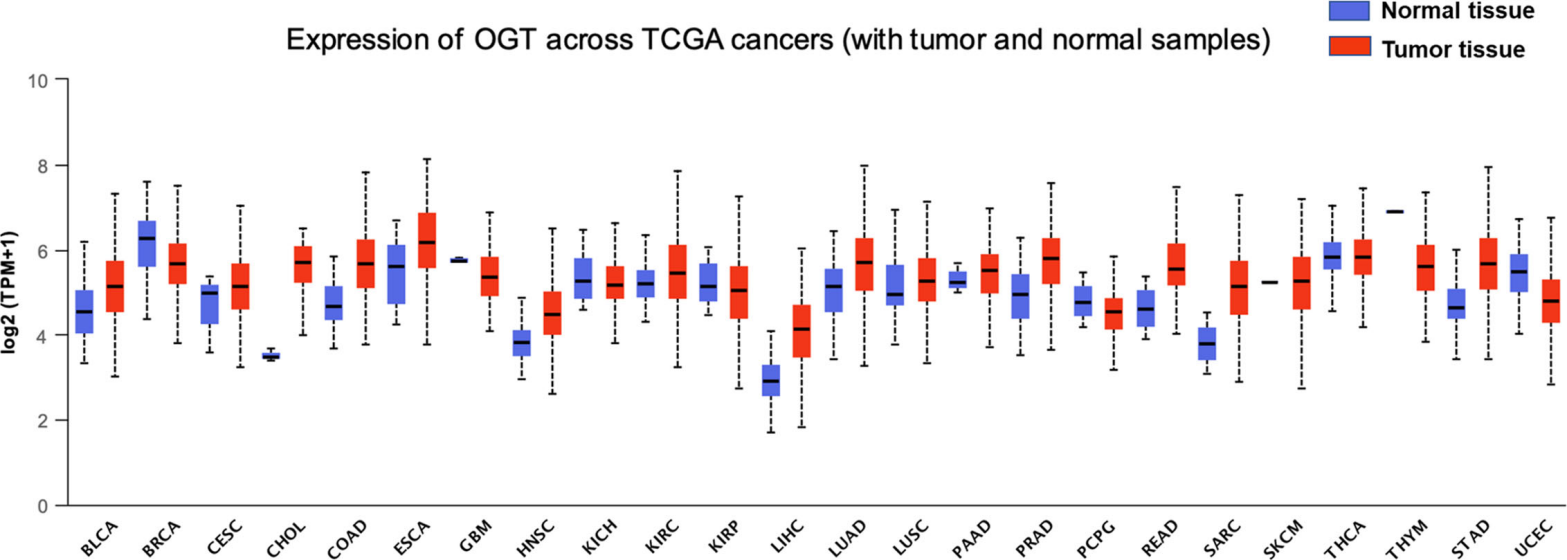
Expression of OGT across multiple cancer types. Pharmacological network analysis was performed by UALCAN (http://ualcan.path.uab.edu) on data extracted from The Cancer Genome Atlas (TCGA). BLCA, bladder urothelial carcinoma, normal tissue = 19, tumor tissue = 408, statistical significance = 2.803800E-03; BRCA, breast invasive carcinoma, normal tissue = 114, tumor tissue = 1,097, statistical significance = 3.12030001836661E-09; CESC, cervical cancer, normal tissue = 3, tumor tissue = 305, statistical significance = 4.214600E-01; CHOL, cholangiocarcinoma, normal tissue = 9, tumor tissue = 36, statistical significance = 2.6247892748188E-12; COAD, chronic obstructive pulmonary disease, normal tissue = 41, tumor tissue = 286, statistical significance = 1.62436730732907E-12; ESCA, esophageal carcinoma, normal tissue = 11, tumor tissue = 184, statistical significance = 6.667600E-02; GBM, glioblastoma, normal tissue = 5, tumor tissue = 156, statistical significance = 2.163600E-01; HNSC, head and neck squamous cell carcinoma, normal tissue = 44, tumor tissue = 520, statistical significance = 2.14550044397299E-10; KICH, kidney chromophobe, normal tissue = 25, tumor tissue = 67, statistical significance = 4.953000E-01; KIRC, kidney renal clear cell carcinoma, normal tissue = 72, tumor tissue = 533, statistical significance = 1.84977999999614E-05; KIRP, kidney renal papillary cell carcinoma, normal tissue = 32, tumor tissue = 290, statistical significance = 7.601200E-01; LIHC, liver hepatocellular carcinoma, normal tissue = 50, tumor tissue = 371, statistical significance = <1E-12; LUAD, lung adenocarcinoma, normal tissue = 59, tumor tissue = 515, statistical significance = 6.66022792472631E-12; LUSC, lung squamous cell carcinoma, normal tissue = 52, tumor tissue = 503, statistical significance = 2.798400E-02; PAAD, pancreatic adenocarcinoma, normal tissue = 4, tumor tissue = 178, statistical significance = 4.355800E-01; PCPG, pheochromocytoma and paraganglioma, normal tissue = 3, tumor tissue = 179, statistical significance = 1.192150E-01; PRAD, prostate adenocarcinoma, normal tissue = 52, tumor tissue = 497, statistical significance = 1.62458935193399E-12; READ, rectum adenocarcinoma, normal tissue = 10, tumor tissue = 166, statistical significance = 8.70579999978638E-07; SARC, sarcoma, normal tissue = 2, tumor tissue = 260, statistical significance = 1.889990E-01; SKCM, skin cutaneous melanoma, normal tissue = 1, tumor tissue = 104, statistical significance = 3.755700E-03; STAD, stomach adenocarcinoma, normal tissue = 3, tumor tissue = 415, statistical significance = <1E-12; THCA, thyroid carcinoma, normal tissue = 59, tumor tissue = 505, statistical significance = 4.631800E-01; THYM, thymoma, normal tissue = 2, tumor tissue = 120, statistical significance = 1.718990E-03; UCEC, uterine corpus endometrial carcinoma, normal tissue = 35, tumor tissue = 546, statistical significance = 1.66160000003579E-06.

*O*-GlcNAcylation harboring many substrates is involved in various cellular processes, including gene transcription regulation, stem cell differentiation, enzyme activity, and protein stability, among others (21–26). In view of the important roles of *O*-GlcNAcylation in multiple fundamental cellular processes, it is unsurprising that imbalanced profiles of OGT/*O*-GlcNAcylation frequently lead to the occurrence of many diseases such as diabetes, neurological disorders, cardiovascular disease, and even cancer (27, 28). In many types of cancer such as breast, prostate, lung, colorectal, and esophageal cancers, higher levels of OGT/*O*-GlcNAcylation are observed (29), suggesting that alterations of the intracellular level of OGT and *O*-GlcNAcylation are tightly associated with tumorigenesis, which might further participate directly or indirectly in the regulation of the biological processes associated with cancer metastasis. For example, the increased levels of OGT/*O*-GlcNAcylation in patients with lung cancer or colon cancer are closely correlated with poor overall survival, as well as the anchorage-independent growth, migration, and invasion ability of lung and colon cancer cell lines (22, 23, 30, 31). Elevated OGT proteins, as well as *O*-GlcNAcylation level, are also found in both breast cancer cells and tumor tissues (24, 25). Further research has revealed that *O*-GlcNAcylation of progesterone receptor (PR) by OGT transcriptionally activates its target genes, and PR-positive breast cancers express higher levels of OGT (26). In addition, 22 of 56 prostate cancer biopsy specimens were found to show increased *O*-GlcNAcylation, which correlated with poor prognosis (28). Furthermore, in prostate carcinoma and bladder cancer cells, while the level of OGT/*O*-GlcNAcylation increased, the level of deglycosylase OGA decreased (32, 33), prompting a dynamic imbalance between OGT and OGA. More in-depth research results confirmed the correlation between the OGT protein level and tumor metastatic progression in prostate cancer cells (32). In addition, downregulation of *O*-GlcNAcylation induced by OGT silencing results in cell cycle arrest, as well as the induction of autophagy and apoptosis, in bladder cancer cells (34, 35). However, in rare cases, *O*-GlcNAcylation is decreased in cancer tissues such as ovarian cancer tissues which harbor high rates of p53 mutations (36). In ovarian cell lines expressing wild-type p53, the high level of OGT/*O*-GlcNAcylation can stabilize the tumor suppressor p53, and stabilized p53 further promotes the acquisition of new pro-oncogenic activities including cell proliferation and metabolic changes, whereas the stabilization of p53 was not detected in cell lines with mutated p53 (36, 37), indicating a role of *O*-GlcNAcylation in regulating ovarian cancer proliferation and progression. Moreover, data of aberrant OGT level in various cancer tissues is also collected and analyzed by UALCAN based on TCGA datasets (**Figure 1**) (38–40). Taken together, the changes in OGT/*O*-GlcNAcylation level directly affect tumor occurrence and progression.

## Role of *O*-GlcNAcylation in Cancer Metastasis

Tumor cells are characterized by high metabolic rates, rapid growth, and high proliferative capacity. They, therefore, exhibit a high energy demand, necessitating anaerobic metabolism within the hypoxic tumor microenvironment (TME). Accumulating evidence indicates that OGT-mediated *O*-GlcNAcylation on a variety of substrates including transcription factors, oncoproteins, and proteins associated with epithelial mesenchymal transition (EMT) promotes tumor metastatic capacity in numerous cancer cells, including those derived from colorectal cancer (CRC), breast cancer, gastric cancer, pancreatic cancer, and cholangiocarcinoma (CCA) (31, 41–45). Of the proteins associated with CCA progression, 21 display *O*-GlcNAcylation sites (46). There are already research data confirming that CRC patients with high *O*-GlcNAcylation are typically diagnosed with greater lymph node metastasis potential (41, 47). Abolishing such modification of actin-binding protein cofilin at Ser108 suppresses the invasive capability of breast cancer cells (48). Moreover, decreasing *O*-GlcNAcylation levels *via* OGT knockdown or microRNA (miRNA; e.g., miR-483 and miR-24-1)-mediated depletion suppresses the growth, migration, and invasive capability of cancer cells (31, 39, 40).

### *O*-GlcNAcylation of Transcription Factors in Cancer Metastasis

**Figure 2:**
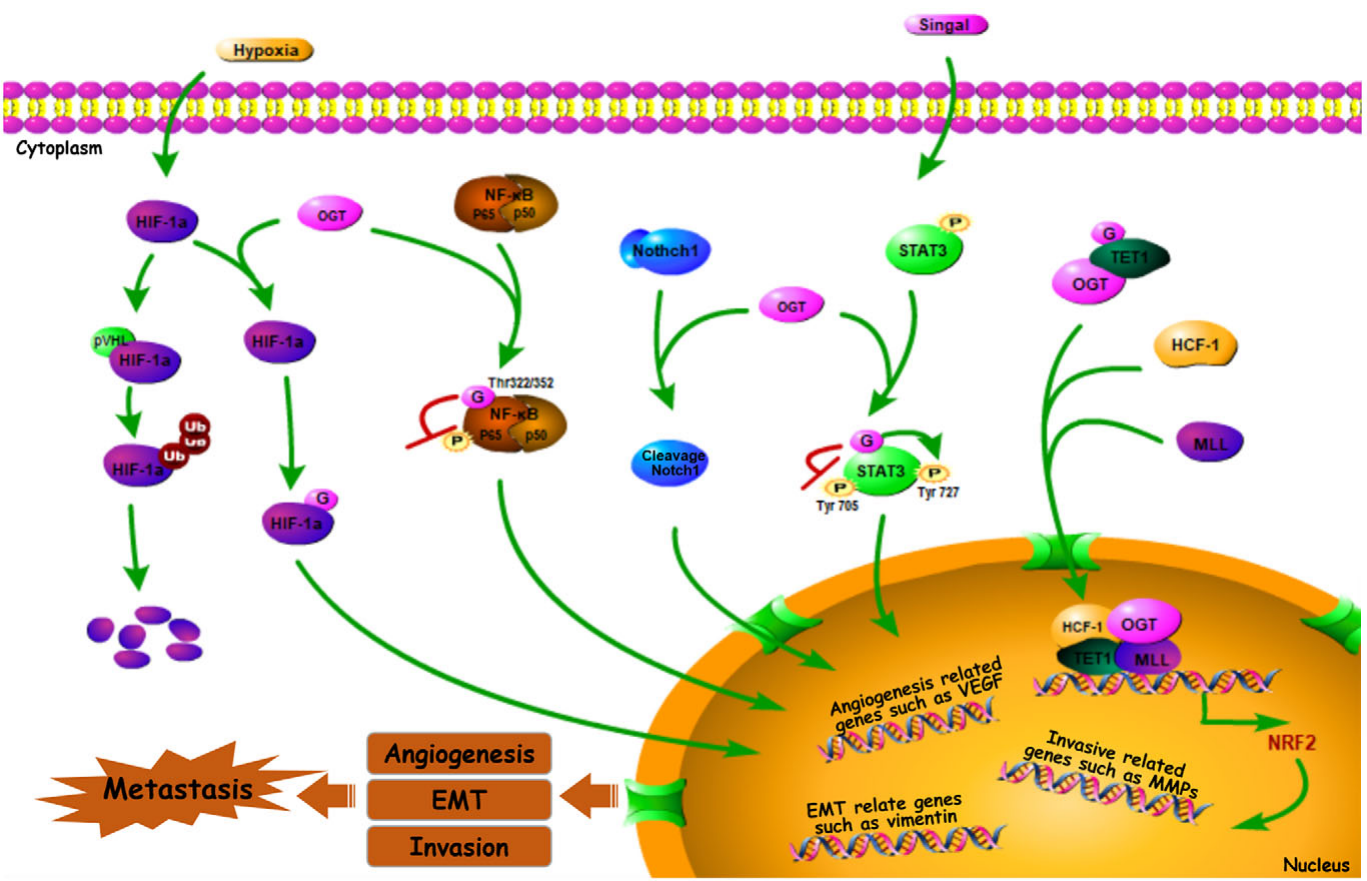
*O*-GlcNAc-modified transcription factors in cancer metastasis. Some *O*-GlcNAc-modified metastasis related transcription factors such as HIF-1α, Notch1, NF-κB, STAT3, Nrf2 can impact cancer metastasis by affecting downstream genes (or proteins) (49–61).

Many genes are involved in the process of cancer metastasis. Therefore, altered global cellular *O*-GlcNAcylation profiles can directly or indirectly impact the expression and activation of transcription factors, and this further change the biological behavior of those regulatory factors (**Figure 2**).

Hypoxia-inducible factor-1α (HIF-1α) is a well-known transcription factor that was originally identified as mediating adaptation to the hypoxic TME (62). It is clear that HIF-1α, the expression of which is induced by hypoxia, further activates the expression of its numerous targets—including matrix metalloproteinases (MMPs), E-cadherin, and transcription factor 3 (TCF3), among others—that enhance cancer metastasis *via* multiple mechanisms, favoring invasion, extravasation, and metastatic niche formation (63–65). OGT stabilizes HIF-1α by suppressing its interaction with von Hippel-Lindau tumor suppressor protein (pVHL), a E3-ubiquitin ligase mediates HIF-1α degradation. Thereby *O*-GlcNAcylation stabilizes HIF-1α and activates its transcriptional activity (49). Meanwhile, decreased OGT expression and *O*-GlcNAcylation level were observed when the protein levels and transactivation of HIF-1α were inhibited (66). This sets up a positive feedback loop, facilitating hypoxic adaptation, which further regulates processes such as immortalization, angiogenesis, invasive capability, and metastasis of breast cancer (49, 50). Indeed, reduced OGT results in a lower angiogenic potential and decreased vascular endothelial growth factor (VEGF) mRNA level in prostate cancer cell line (32). However, VEGF-mediated angiogenesis within tumors can be driven by HIF-1α activation (47). Furthermore, HIF-1α-induced VEGF upregulation promotes retinal angiogenesis in rats (67). Suggesting that by mediating the stabilization and activation of HIF-1α, OGT regulates HIF-1α target genes and functions in angiogenesis, as well as cancer metastasis (56).

Notch receptor 1 (Notch1), a type 1 trans-membrane receptor, is a key regulator of tumor angiogenesis and metastasis. It exhibits sustained activation in pre-metastatic lesions, which promotes migration in various types of tumor cells, including those derived from CRC, lung cancer, and melanoma (68, 69). In addition, Notch1 signaling plays a critical role in metastasis, including metastatic initiation in medulloblastoma and the promotion of highly-penetrant metastases in CRC (70, 71). Recently, it was demonstrated that OGT can *O*-GlcNAcylate Notch1, a process enhanced by glucosamine, resulting in the cleavage and nuclear translocation of Notch1 (51), thereby regulating the transcription of target genes, suggesting the importance of Notch1 transcriptional activity in cancer metastasis by modulating its *O*-GlcNAcylation.

Based on one report, nuclear factor κB (NF-κB), a transcription factor, is not only critically involved in the inflammatory response (including regulating IL-1β and IL-6 expression), but also contributes to tumor hematologic and lymphatic metastases, suggesting the correlation between NF-κB signal pathway and cancer metastasis (65). In breast cancer cells, one of the most common NF-κB dimeric forms, RELA (p65)/p50, can be *O*-GlcNAcylated at Thr322 and Thr352 residues of p65, which competitively inhibits p65 Ser536 phosphorylation, thus facilitating activated NF-κB-mediated gene transcription (52–54). Further, inflammation has timing- and context- specific roles during tumorigenesis and progression to cancer. For example, while NF-κB *O*-GlcNAcylation promotes MMP-mediated migration and invasive capability of CCA cells (55), NF-κB p65 *O*-GlcNAcylation downregulates C-X-C chemokine receptor 4 (CXCR4) to inhibit cervical cancer (CESC) cell metastasis to the lungs (56), and NF-κB activation-mediated upregulation of inducible nitric oxide synthase (iNOS) modulates immune suppression and tumor progression (57, 58). Activation of NF-κB *via O*-GlcNAcylation, therefore, modulates the expression of a variety of downstream genes involved in both tumor suppression and progression (72), indicating a role of OGT in regulating cancer metastasis by changing the NF-κB activation through its *O*-GlcNAc modification.

The transcription factor signal transducer and activator of transcription 3 (STAT3) is constitutively activated in tumors of different origins. Phosphorylation can activate STAT3, resulting in its translocation to the nucleus to regulate gene expression, further enhancing tumor angiogenic and invasive capability (73). For instance, phosphorylated STAT3 promotes proliferation and metastasis in epithelial ovarian cancer (74). Cross-talk between STAT3 *O*-GlcNAcylation and phosphorylation also occurs, with the former inhibiting the latter (75–77). Phosphorylation targets STAT3 residues, Tyr705 and Ser727, and these two modifications demonstrate a negative relationship to maintain its activity (78). Whereas STAT3 *O*-GlcNAcylation promotes Tyr705 phosphorylation, it inhibits Ser727 phosphorylation (59, 60), thereby enhancing metastasis by regulating STAT3 signaling and target gene transcription.Nuclear factor erythroid 2-related factor 2 (Nrf2) is another transcription factor, the activation of which plays a critical role in sustained angiogenesis, tumor invasion and metastasis (79). For example, activated Nrf2 stabilizes BTB domain and CNC homolog 1 (BACH1), accelerating lung cancer metastasis (80). Similarly, Nrf-2 activation promotes CRC and hepatic carcinoma metastasis (81). Nrf-2 activation is likely modulated by OGT, cause in *Caenorhabditis elegans*, the ortholog of human Nrf-2, is *O*-GlcNAcylated at Ser470 and Thr493 (82). Moreover, Nrf-2 transcriptional level is initiated when OGT is recruited at the promoter region by ten-eleven translocation 1 (TET1), and form a complex with host cell factor 1(HCF1) and mixed-lineage leukemia (MLL) (61).

### *O*-GlcNAcylation of E-Cadherin in Cancer Metastasis

Cancer cells undergoing EMT acquire the characteristics of aggressive, more invasive, stem-like features, with increased ability for cell migration, invasion and metastasis (83). In cancer cells and embryonic stem cells, *O*-GlcNAc-modification frequently facilitates the occurrence of EMT. For example, OGT is required for the induction and maintenance of EMT in NSCLC (84). In addition, hyper-*O*-GlcNAcylation contributes to the EMT of EC (85). The cell surface protein E-cadherin mediates cell-cell interactions, which is directly correlated with cancer cell adhesiveness, and this therefore mediates the invasive and metastatic capabilities of cells (86). While high levels of soluble E-cadherin in ovarian cancer-associated ascitic fluid promote tumor angiogenesis (87), decreased surface E-cadherin levels promote metastasis of breast cancer cells and lung adenocarcinoma cells (88, 89). Increased OGT expression and higher global *O*-GlcNAcylation levels suppress E-cadherin expression, thereby promoting breast cancer metastasis to the lungs (25). This inverse relationship between E-cadherin expression and metastatic potential also exists in ovarian cancer and CRC cells (90, 91). Notably, transcriptional expression of E-cadherin can be regulated by upstream proteins. For instance, Snail as an E-cadherin repressor can stabilize E-cadherin *via* Ser112 *O*-GlcNAcylation and enhance the migration and invasive capability of cancer cells (92). In addition, the cytoskeletal protein vimentin is a substrate of OGT, and the stabilization of E-cadherin is regulated by the *O*-GlcNAcylation status of vimentin (93, 94). Moreover, E-cadherin can be directly *O*-GlcNAcylated in breast cancer cells during drug-induced apoptosis, and this modification inhibits its transport to the cell surface, thereby decreasing cell-cell interactions and promoting EMT. Decreased surface E-cadherin levels increase infiltrative capacity, and cancer cell proliferation and survival are simultaneously decreased (95). *O*-GlcNAcylation of EMT-Related Proteins in Cancer Metastasis

During EMT, reduced E-cadherin expression and elevated snail, vimentin, fibronectin, and N-cadherin expression levels can be observed, thus these proteins are considered EMT markers (96). Beyond these markers, many EMT-related proteins including transcriptional factors are involved in the process of EMT. Receptor for activated protein kinase C (RACK1), encoded by GNB2L1, is a scaffold protein. (97). RACK1 induces EMT, further promotes the progression of esophageal squamous cell carcinoma (ESCC) and glioma (98, 99). Moreover, *O*-GlcNAcylation of RACK1 by OGT stabilizes RACK1, and results in a reduction of N-cadherin and upregulation of E-cadherin, indicating the induction of EMT and suppression of metastasis in chemoresistant gastric cancer (79, 100, 101). Numerous transcriptional factors including HIF-1α, Notch1, NF-κB have a critical role in EMT procession (102). For example, STAT3 regulates the expression of mesenchymal-related molecules including vimentin, the inhibition of which suppresses EMT-mediated lung cancer cell invasion (103). By regulating these transcriptional factors, the role of *O*-GlcNAcylation in EMT could be understood.

### *O*-GlcNAcylation of MMPs in Cancer Metastasis

The MMP family plays a critical role in cancer cell migration. For example, MMP-9 overexpression is often observed across numerous malignant tumor types, and MMP-9 has been investigated for its potential as a cancer biomarker (104). Decreased global cellular *O*-GlcNAcylation levels result in decreased MMP-9 mRNA and protein levels, concurrently decreasing migration, invasive, and metastatic capability of gastric and EC cells (105, 106). Sirtuin1 (SIRT1) is a histone de-acetylase and *O*-GlcNAcylation of SIRT1/Ser549 promotes its enzymatic activity (107). Decreasing *O*-GlcNAcylation of this protein *via* OGT inhibition or knockdown in breast cancer cells increases both SIRT1 level and activity, thereby regulating forkhead box M1 (FOXM1), MMP-2, and MMP-9 protein level, and modulating breast cancer cell invasive and metastatic capability *in vitro* and *in vivo* (42). Via MMP targeting, *O*-GlcNAcylation plays an important role in cancer metastasis (47, 95, 100, 101, 106–121).

In summary, based on substrates of OGT, as well as their downstream effectors, which have key roles in regulating hypoxia, gene transcription, EMT, and metastasis, O-GlcNAcylation significantly modulates cancer progression.

## Interplay Between *O*-GlcNAcylation and Other PTMs in Cancer Metastasis

Various PTMs of intracellular proteins rely on epigenetic regulatory enzymes with different catalytic functions. Generally, different PTMs often coordinate with each other to adapt to the process of complex biological functions in cells. *O*-GlcNAcylation is no exception. There has been much evidence confirming the interactions between *O*-GlcNAcylation and other PTMs. As a typical example (17, 122–127), both *O*-GlcNAcylation and phosphorylation occur on Ser/Thr residues of substrate proteins, and extensive crosstalk between two PTMs through mutual inhibition of the same or nearby residues has been identified (108). 6-phosphofructo-2-kinase/fructose-2,6-bisphosphatase 3 (PFKFB3), a glycolytic regulator, can be *O*-GlcNAcylated and phosphorylated at Ser172, and the competition between these two PTMs regulates the function of PFKFB3 in promoting nasopharyngeal carcinoma and gastric cancer proliferation, as well as migration (109–111). The enhancer of zeste homolog (EZH2) is responsible for H3K27me3, which promotes the metastasis of cancers such as melanoma and breast cancer (112, 113). *O*-GlcNAcylation at Ser729 of EZH2 plays a key role in maintaining the stabilization and methylation activity of its target protein (114, 118). Further, ubiquitination-mediated degradation of EZH2 suppresses breast cancer invasion and metastasis (119), and *O*-GlcNAc-modified EZH2 could reverse this degradation. EZH2 is stabilized by OGT *via O*-GlcNAcylation and promotes EMT and metastasis of CRC (41). The critical roles of histone deacetylases (HDACs) in tumorigenesis and tumor progression have been widely studied. Among them, HDAC1 and SIRT1 were identified as being *O*-GlcNAcylated at certain residues, and *O*-GlcNAcylation on specific residues further promotes the histone deacetylase activity of HDAC1 and SIRT1 (107, 116). In breast cancer cells, Nrf1 can be stabilized by OGT through *O*-GlcNAcylation at Ser448 and Ser451, a modification that suppresses the ubiquitin-proteasome mediated degradation of Nrf1. In contrast, reduced expression of Nrf1 suppresses its invasion and migration ability (115, 128). In summary, crosstalk between *O*-GlcNAcylation and other PTMs plays critical roles in regulating cancer metastasis.

## Small Molecules That Target *O*-GlcNAcylation

Tumorigenesis and tumor progression are often accompanied by higher *O*-GlcNAcylation, which likely drives a range of oncogenic adaptations made by cancer cells, including rapid proliferation. Therefore, inhibiting global *O*-GlcNAcylation levels may also be an effective anti-cancer approach. In line with this, reducing intracellular OGT levels has been shown to inhibit the growth of lung cancer cells (23). A similar phenomenon was revealed in bladder cancer cells and renal cell carcinoma (RCC). Knocking down OGT results in cell cycle arrest as well as induction of autophagy and apoptosis (34, 35, 117). Considering the critical function of aberrant *O*-GlcNAcylation in cancer progression and metastasis which has been summarized previously herein, it is likely that downregulation of hyper *O*-GlcNAcylation *via* OGT inhibition might not only slow cancer proliferation, but also cancer metastasis.

In light of the findings that high levels of *O*-GlcNAcylation and OGT can affect multiple targets and signaling pathways during tumorigenesis, efforts are being made to find small molecules that can inhibit the activity of OGT. By rebalancing global *O*-GlcNAcylation profiles or targeting specific *O*-GlcNAcylated proteins, small molecules targeting OGT have been identified as exhibiting anti-cancer therapeutic potential. For example, miRNA-24, miRNA-101, and miRNA-483, all of which decrease OGT transcription, have been shown to inhibit the invasive ability of breast cancer, CRC, and gastric cancer, respectively (40, 41, 100). Similarly, ST045849, an OGT inhibitor, suppresses prostate cancer cell proliferation *via* metabolic reprogramming, and has been shown to inhibit hepatocellular carcinoma (HCC) cell proliferation (101, 120). Another OGT inhibitor, OSMI-1, developed *via* high-throughput screening, inhibits protein *O*-GlcNAcylation (121) and decreases tumor volume (129). Furthermore, the OGT inhibitor OSMI-2 decreases global chromatin *O*-GlcNAcylation and inhibits the proliferation of prostate cancer cells as a single drug. This suppression is also observed in organoids derived from patients with metastatic prostate cancer but not normal prostate cells, when OSMI-2 was combined with a CDK9 inhibitor (130, 131). In addition, Ac-5SGlcNAc, an OGT inhibitor that decreased global *O*-GlcNAcylation, but not N-glycosylation or N-glycosylation, suppresses the proliferation of pancreatic and breast cancer cells (54, 122, 132). Ac-5SGlcNAc treatment also blocks serum-stimulated cyclin D1 synthesis during the G0/G1 transition of breast cancer cells, suggesting that the role of OGT inhibitors in regulating the cell cycle further affects cell proliferation (123). Novel OGT-targeting small molecules are regularly identified. For instance, BZX2, OSMI-3, OSMI-4, L01, and ES1 have been identified as OGT inhibitors, but their broader biological impact is yet to be explored (124–127). Given the critical roles of OGT, such small molecule inhibitors may contribute substantially towards clarifying the function of OGT in cancer metastasis, and may be developed as clinically applicable anti-cancer therapeutic agents that can be used alone or in combination with other drugs (**Table 2**). However, considering the key roles of OGT in normal cell processes (e.g., energy metabolism), small molecule inhibitors of OGT might also impact normal physiology. Thus, studies focused on correcting aberrant *O*-GlcNAcylation to normal levels will need, to prevent or mitigate such off-target and potentially adverse effects.

**Table 2:** Small molecules targeting *O*-GlcNAcylation in cancer progression.

Small Molecules	Structure	Cancer	Mechanisms	Reference(s)
Ac-5SGlcNAc	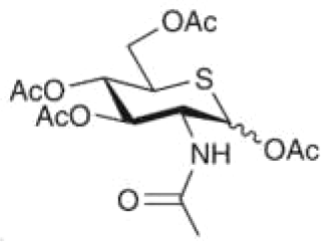	CRC	Delayed cell proliferation and decreased migration.	(122, 133),
		PDAC	Targets OGT, promoting apoptosis of PDAC	(54, 122),
ST045849	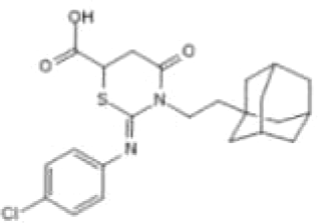	prostate cancer	Targets OGT, and suppresses proliferation of prostate cancer and HCC.	(33, 34, 101, 120),
		HCC		
OSMI-1	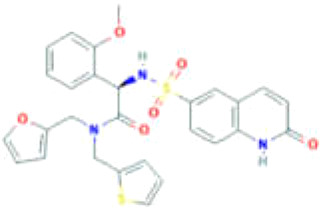	prostate cancer	Suppresses *O*-GlcNAcylation levels and inhibits proliferation of prostate cancer cells.	(36)
		pancreatic cancer	Inhibits cancer cell proliferation	(134)
		endometrial cancer	Inhibition of cell proliferation and migration	(85)
		mouse hepatoma cell	Decreased cell invasive behavior in high metastatic Hca-F cells	(39)
OSMI-2	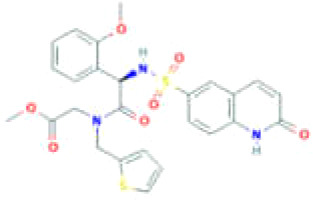	prostate cancer	Inhibits OGT activity, promoting proliferation of prostate cancer.	(37, 130, 131),

*CRC, colorectal cancer; HCC, hepatocellular carcinoma, PDAC, human pancreatic ductal adenocarcinoma

## Conclusions and Perspectives

**Figure 3:**
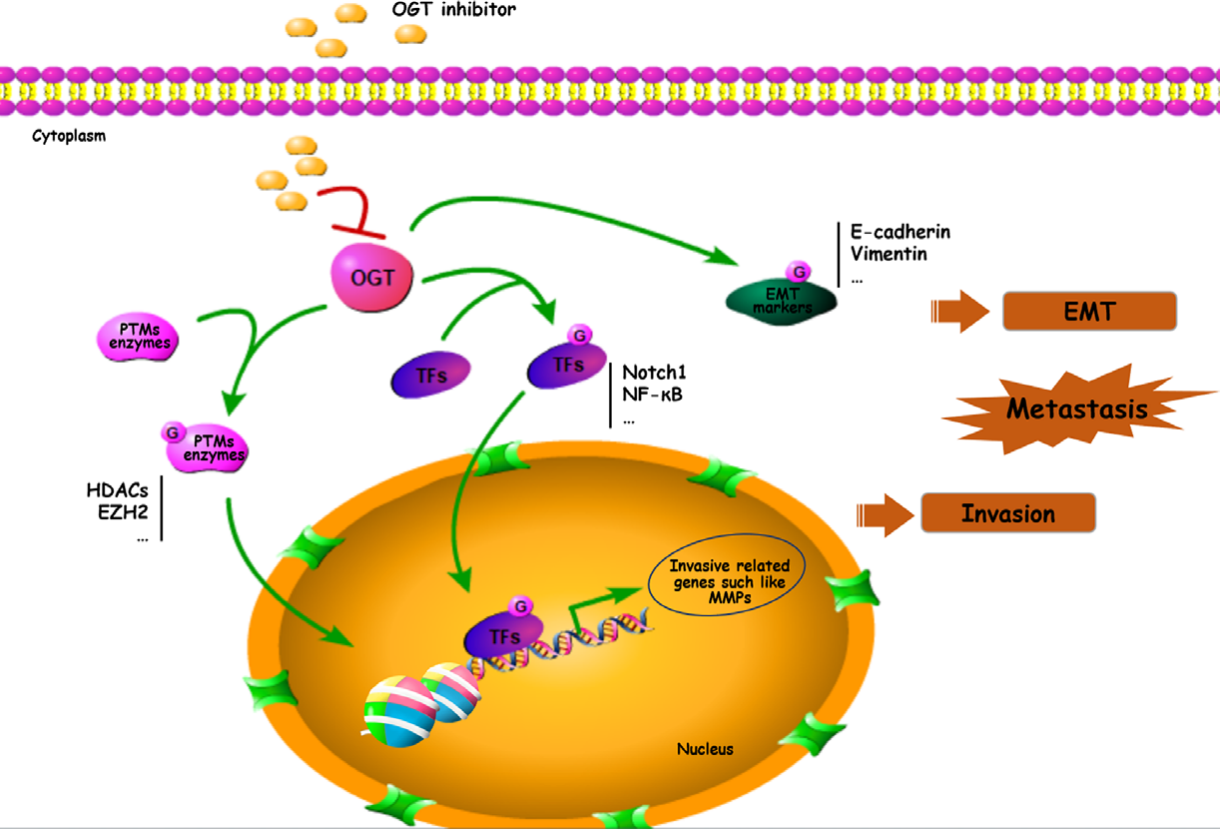
Important roles of *O*-GlcNAcylation on EMT/PTMs-related proteins in cancer metastasis. Many proteins including transcriptional factors, EMT related proteins, MMPs and PTMs enzymes can be *O*-GlcNAc-modified and participated in cancer metastasis. TFs, transcriptional factors.

*O*-GlcNAcylation is implicated in various fundamental cellular processes *via* the regulation of gene transcription, metabolism, and various signaling pathways. Several potential mechanisms by which OGT-mediated *O*-GlcNAcylation of substrate proteins modulates cancer progression include the following cellular processes: (1) creating recognition sites for recruitment to initiate cascades leading to the activation of downstream effectors, (2) cross-talk with PTMs to modulate substrate stabilization and activation, (3) integration of EMT/transcription factors/metastasis-associated protein activities, and (4) directing cancers towards metastasis *via* high levels of protein *O*-GlcNAcylation (**Figure 3**).

Elucidating the functional mechanisms through which *O*-GlcNAcylation promotes cancer metastasis will provide a theoretical basis for future rational research. Considering the close relationship between *O*-GlcNAcylation and cancer progression-associated pathways, small molecules targeting OGT may have potential as anti-cancer therapies, especially in the inhibition of metastasis. In particular, the anti-cancer activities of more specific OGT inhibitors, alone or in combination with other drugs, as well as the side effects should be further investigated.

## Author Contributions

DW, JJ, and DL participated in writing, editing, and making figures. ZQ and HL read and approved the final manuscript. All authors contributed to the article and approved the submitted version.

## Funding

National Natural Science Foundation of China, (grant NO. 81903876, 81803680, 81973712, 81973468, 81803649). Jilin Province Traditional Chinese Medicine Technology Project (grant No. 2019051, 2020041).

## Conflict of Interest

The authors declare that the research was conducted in the absence of any commercial or financial relationships that could be construed as a potential conflict of interest.

## Abbreviations

CCA, cholangiocarcinoma; CESC, cervical cancer; CRC, colorectal cancer; CXCR4, C-X-C chemokine receptor 4; EC, esophageal cancer; EMT, epithelial-mesenchymal transition; EZH2, zeste homolog; HBP, hexosamine biosynthetic pathway; HCC, hepatocellular carcinoma; HDAC, histone deacetylase; HIF-1α, hypoxia-inducible factor 1 alpha; H3K27, histone 3 lysine 27; iNOS, Inducible Nitric Oxide Synthase; MMPs, Matrix metalloproteinases; NF-κB, Nuclear factor κB; Notch1, notch receptor 1; Nrf, Nuclear factor erythroid 2-related factor; *O*-GlcNAc, *O*-linked β-N-Acetylglucosamine; *O*-GlcNAcylation, *O*-linked β-N-Acetylglucosamine modification, *O*-GlcNAc modification; OGT, *O*-GlcNAc transferase; OGA, O-GlcNAcase; PFKFB3, 6-phosphofructo-2-kinase/fructose-2,6-bisphosphatase 3; PTM, post-translational modification; RACK1, Receptor for activated protein kinase C; RCC, renal cell carcinoma; Ser, Serine; SIRT1, sirtuin1; STAT3, Signal transducer and activator of transcription 3; Thr, Threonine; THYM, thymoma; TME, tumor microenvironment; UDP-GlcNAc, Uridine diphospho-N-acetylglucosamine; VEGF, vascular endothelial growth factor.
